# Clinical application of injectable growth factor for bone regeneration: a systematic review

**DOI:** 10.1186/s41232-019-0109-x

**Published:** 2019-10-22

**Authors:** Yutaka Kuroda, Toshiyuki Kawai, Koji Goto, Shuichi Matsuda

**Affiliations:** Department of Orthopaedic Surgery, Graduate School of Medicine, Kyoto University, Shogoin, Kawahara-cho 54, Sakyo-ku, Kyoto, 606-8507 Japan

**Keywords:** Bone regeneration, Growth factor, Cell proliferation, Gelatin hydrogel, Fibroblast growth factor, Clinical trial, Osteonecrosis, Drug delivery system, Tissue engineering

## Abstract

Bone regeneration has been the ultimate goal in the field of bone and joint medicine and has been evaluated through various basic research studies to date. Translational research of regenerative medicine has focused on three primary approaches, which are expected to increase in popularity: cell therapy, proteins, and artificial materials. Among these, the local injection of a gelatin hydrogel impregnated with the protein fibroblast growth factor (FGF)-2 is a biomaterial technique that has been developed in Japan. We have previously reported the efficacy of gelatin hydrogel containing injectable FGF-2 for the regenerative treatment of osteonecrosis of the femoral head. Injectable growth factors will probably be developed in the future and gain popularity as a medical approach in various fields as well as orthopedics. Several clinical trials have already been conducted and have focused on this technique, reporting its efficacy and safety. To date, reports of the clinical application of FGF-2 in revascularization for critical limb ischemia, treatment of periodontal disease, early bone union for lower limb fracture and knee osteotomy, and bone regeneration for osteonecrosis of the femoral head have been based on basic research conducted in Japan. In the present report, we present an extensive review of clinical applications using injectable growth factors and discuss the associated efficacy and safety of their administration.

## Background

The local administration of signaling molecules to act as stimulators has been considered an ideal method for tissue regeneration because of increased cancer risk from repeated systemic exposure. However, morphogenic proteins, such as growth factor (GF), have a relatively short effective half-life at the operation site due to poor protein stability. GFs, in their native form, have several limitations, such as limited interactions with the surrounding extracellular matrix and biologic instability to withstand heat or varying pH conditions. The limited success of GF-based therapy in clinical practice is also associated with the lack of appropriate delivery methods [1–3]. Therefore, over past decades, a considerable number of studies have been conducted on recombinant technology of GFs and drug delivery systems (DDS) using various carriers. In particular, natural polymers such as collagen, gelatin, fibrinogen, hyaluronic acid, and chitosan, among others, have been a research target for scaffold-based DDS because they are often soluble in water and are relatively harmless to the bioactivity of GFs. The practical clinical application of GFs in regenerative medicine has advanced based on these combinatorial protein engineering approaches.

Historically, the nerve GF (NGF) reported by Levi-Montalcini et al. was the first cell GF to be identified [4]. GFs can be defined as soluble-secreted signaling polypeptides that regulate undifferentiated cell proliferation and differentiation to increase or decrease specific cell populations by binding to receptors and transmitting intracellular signals. In the human body, GFs are generally used to trigger the activity of endogenous proteins that promote cell proliferation and differentiation. These factors exhibit various functions in the regulation of cytological and physiological processes by binding to receptor proteins on the surface of target cells, acting as intercellular signal transducers. Cytokines are substances that enable communication among cells via immune system fluids and the hematopoietic system. Conversely, the function of GFs has also been studied in the research of solid tissues. Some GFs act as cytokines or hormones and promote cell differentiation and maturation, rather than cell proliferation [5]. While some cytokines, such as granulocyte colony-stimulating factor and granulocyte–macrophage colony-stimulating factor, are GFs, other cytokines, such as Fas ligand, inhibit cell proliferation or induce cell death (apoptosis). GFs can be classified into several families according to their structural and evolutional characteristics. Most cytokines are peptides or proteins, which are thought to be crucial during cell development and differentiation, with research on their receptors and relationships with carcinogenic mechanisms being actively underway. GFs include NGFs, which promote differential growth such as that of sympathetic ganglion nerve cells; epidermal growth factors which promote the proliferation and differentiation of epithelial cells; fibroblast growth factors (FGFs); hepatocyte growth factors; and bone morphogenetic proteins (BMP). GFs involved in the regulation of bone metabolism include FGF, BMP, transforming growth factor beta (TGF-β), platelet-derived growth factor (PDGF), vascular endothelial growth factor (VEGF), and insulin-like growth factor (IGF) [6].

Common limitations of all GFs include their extremely brief periods of biological activity and specified durations of local effective concentrations. Thus, DDS technology, enabling the sustained release of GFs, is essential for tissue regeneration. Among the natural polymers expected to be effective scaffolds, gelatin-based hydrogels demonstrated the controlled release of GFs at the target site over an extended time period. Gelatin hydrogels are cross-linked hydrophilic polymer networks providing stability and cross-communication with GFs [1, 3]. Moreover, Japanese researchers are currently developing injectable hydrogels containing GFs [7–9], which can be administered using minimally invasive techniques rather than conventional open surgeries. In the present review, we focus on GFs with osteogenic, angiogenic, and tissue repair actions, summarize the examples of clinical applications of injectable GFs, and discuss their practical applications (Table 1).

**Table 1:** Clinical trials using injectable growth factor

Target disease, year, author	Growth factor	Carrier, product characters	Number of patients	Results	Severe adverse events
Critical limb ischemia, 2007, Marui et al. [10]	rhFGF-2, 200 μg	Gelatin hydrogel, injectable syringe	7	Vascular regeneration	No severe adverse events
Knee osteotomy for osteoarthritis, 2007, Kawaguchi et al. [11]	rhFGF-2; 200, 400, 800 μg	Gelatin hydrogel, injectable syringe	57	Dose-dependent early bone healing	Recovered without problem
Periodontitis; 2008, 2011; Kitamura M et al. [12, 13]	rhFGF-2; 0.03%, 0.1%, 0.3%	Hydroxypropylcellulose, injectable syringe	59	Periodontal regeneration	Recovered without problem
Tibial fracture, 2010, Kawaguchi et al. [14]	rhFGF-2; 800, 2400 μg	Gelatin hydrogel, injectable syringe	47	Early bone healing	Recovered without problem
Glucocorticoid-resistant sudden sensorineural hearing loss, 2010, Nakagawa et al. [15]	rhIGF-1, 10 mg	Gelatin hydrogel, injectable	25	Hearing improvement	Recovered without problem
Sudden deafness refractory to systemic corticosteroid treatment, 2014, Nakagawa et al. [16]	rhIGF-1, 300 μg	Gelatin hydrogel, injectable	62	Hearing improvement	Recovered without problem
Osteonecrosis of the femoral head, 2011, Kuroda et al. [17]	rhFGF-2, 800 μg	Gelatin hydrogel, sheet, injectable	10	Nine of ten patients revealed bone regeneration	Recovered without problem
Osteonecrosis of the femoral head, Completed March 2019, Kuroda et al.	rhFGF-2, 800 μg	Gelatin hydrogel, injectable syringe	64	Under evaluation	Under evaluation

*rh* recombinant human, *FGF* fibroblast growth factor, *IGF* insulin-like growth factor

### The advent of the gelatin hydrogel

**Fig. 1:**
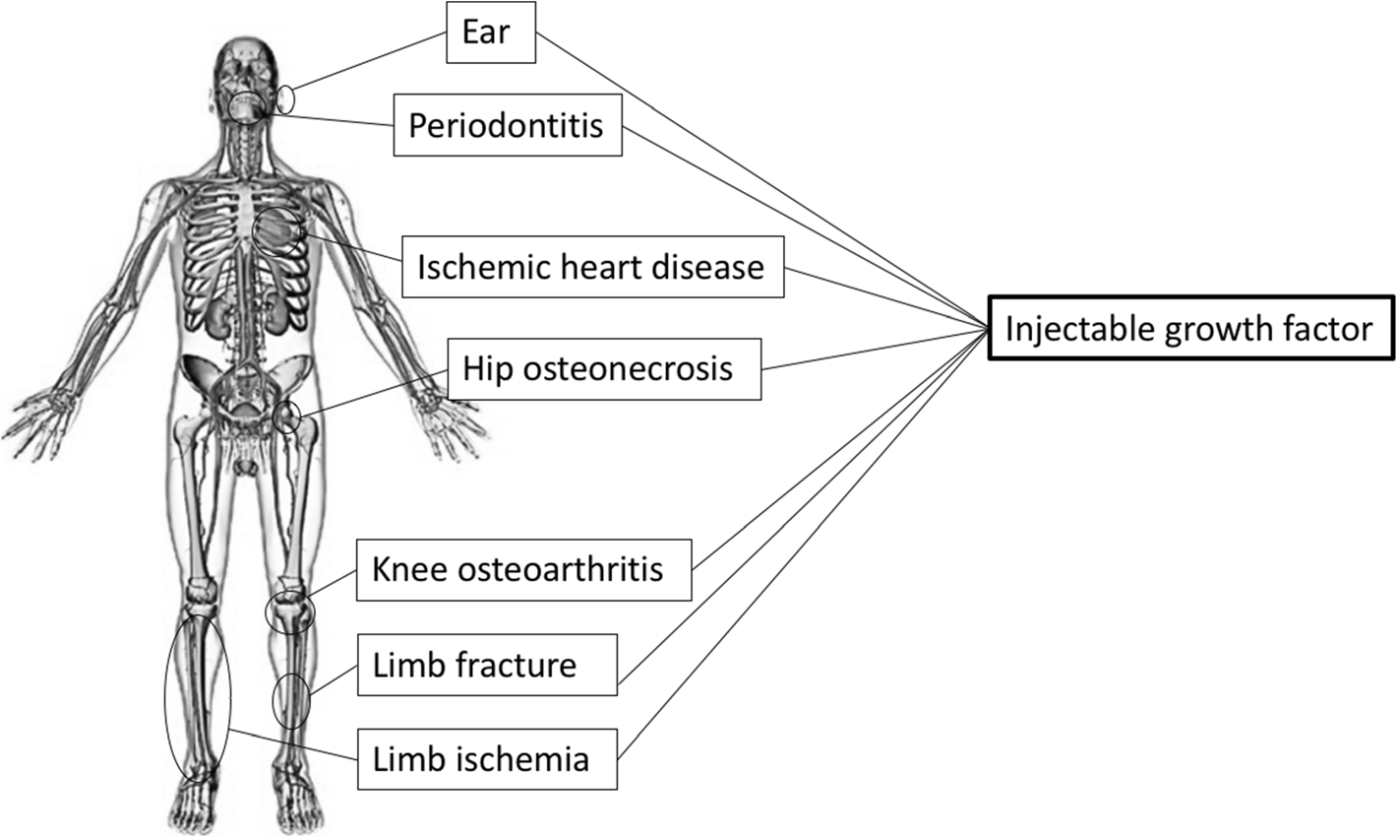
Human figure showing where clinical applications of injectable growth factor are used. Injectable growth factor therapy is actually being performed from the head to toe

**Fig. 2:**
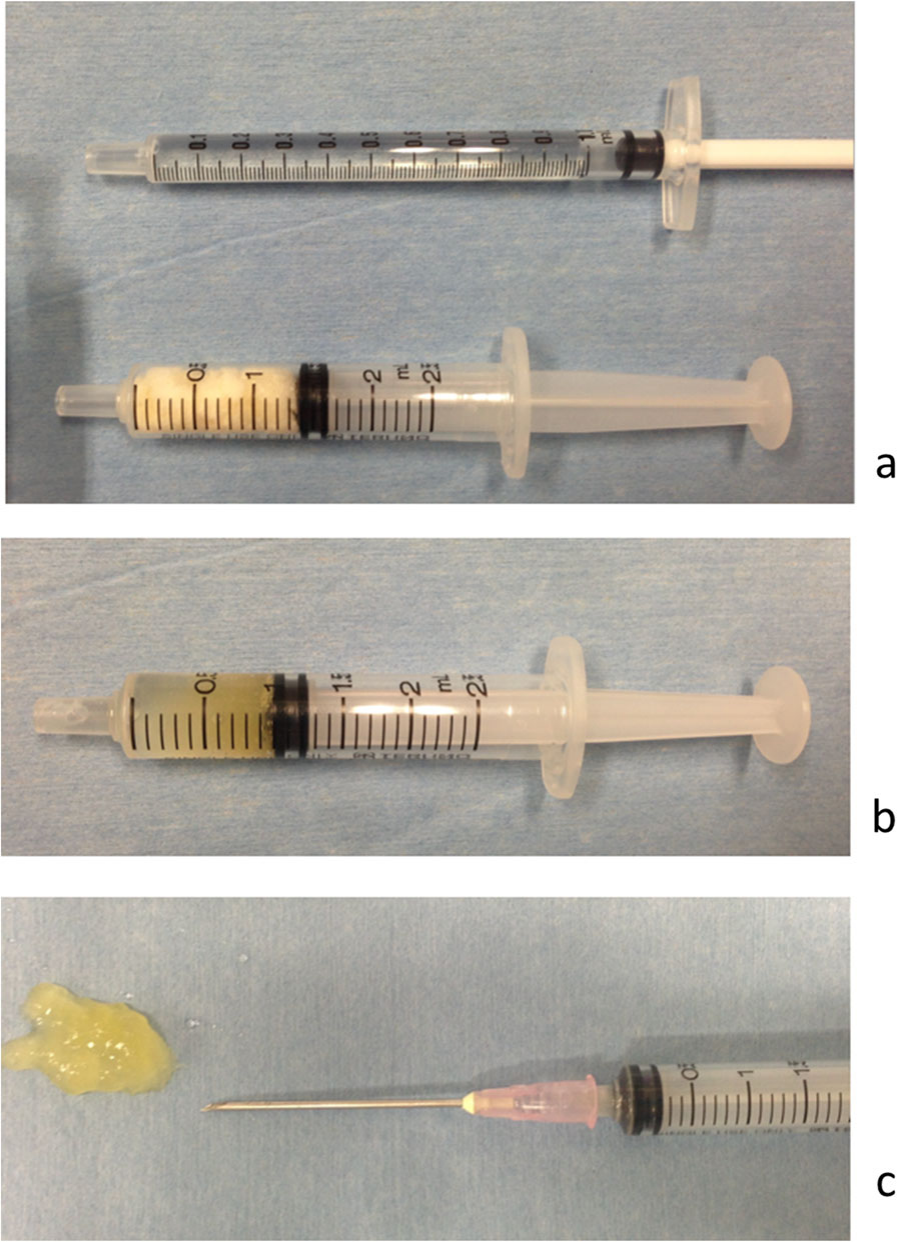
Injectable gelatin hydrogel containing growth factor. The growth factor solution is impregnated in gelatin hydrogel to create a gel-form that can be percutaneously injected using a syringe. **a** Preparation of the growth factor solution (upper) and the freeze-dried gelatin (lower). **b** A gel-form of growth factor-impregnated gelatin hydrogel inside the syringe. **c** Injected gel-form containing growth factor

Gelatin hydrogel is a bioabsorbable material that is produced by the chemical crosslinking of gelatin. It contains various solidified proteins, which have preserved bioactivity through physiochemical (mainly electrostatic) interactions. The use of cross-linked gelatin has enabled the immobilization and regulation of the local release of GFs [1, 3]. Tabata et al. reported that the release of GFs from the hydrogel at the site of implantation was controllable for more than 2 weeks, a period that correlates strongly with the patterns of in vivo GF release and hydrogel degradation [8]. A gelatin sample with an isoelectric point of 5.0 was isolated from bovine bone through an alkaline process. The gelatin hydrogel was prepared through the glutaraldehyde crosslinking of gelatin at 4 °C for 12 h. The processed hydrogels were soaked in a glycine aqueous solution for 3 h to block the residual aldehyde groups of the hydrogels. The hydrogels were then rinsed three times with distilled water at room temperature. The homogenates of gelatin hydrogels were passed through sieves with different mesh sizes and collected as microspheres with diameters ranging from 50 to 100 μm and freeze-dried [7–9, 18, 19]. In this hydrogel system, the GF immobilized in the acidic gelatin hydrogel is released only when the hydrogel is degraded to generate water-soluble gelatin fragments. Gelatin hydrogels have been modified to be more acidic or more basic in order to increase ionic interactions with oppositely charged GFs [1]. The controlled release of FGF-2 from a negatively charged gelatin hydrogel, or BMP-2 from a positively charged one, has respectively shown improved regeneration of cartilage and bone [18, 19]. Thanks to the advent of the gelatin hydrogel, several research studies on cell GFs and gelatin hydrogels containing recombinant human (rh) GF are currently underway. Furthermore, the gelatin hydrogel can be modified into a sheet, disk, or granular forms, enabling broad applications. Especially, the injectable hydrogels containing GFs have an even more relevant clinical application as these can be administrated using minimally invasive techniques. Minimally invasive procedures using the injectable GF has several advantages over conventional procedures, such as less operative trauma, complications, and adverse events. The development of these products has been done with their clinical application in mind (Fig. 1). In fact, these injectable GF hydrogels are packaged in a convenient and ready-to-use kit consisting of a syringe containing the freeze-dried gel and GF solution (Fig. 2).

### Fibroblast growth factor (FGF)

**Fig. 3:**
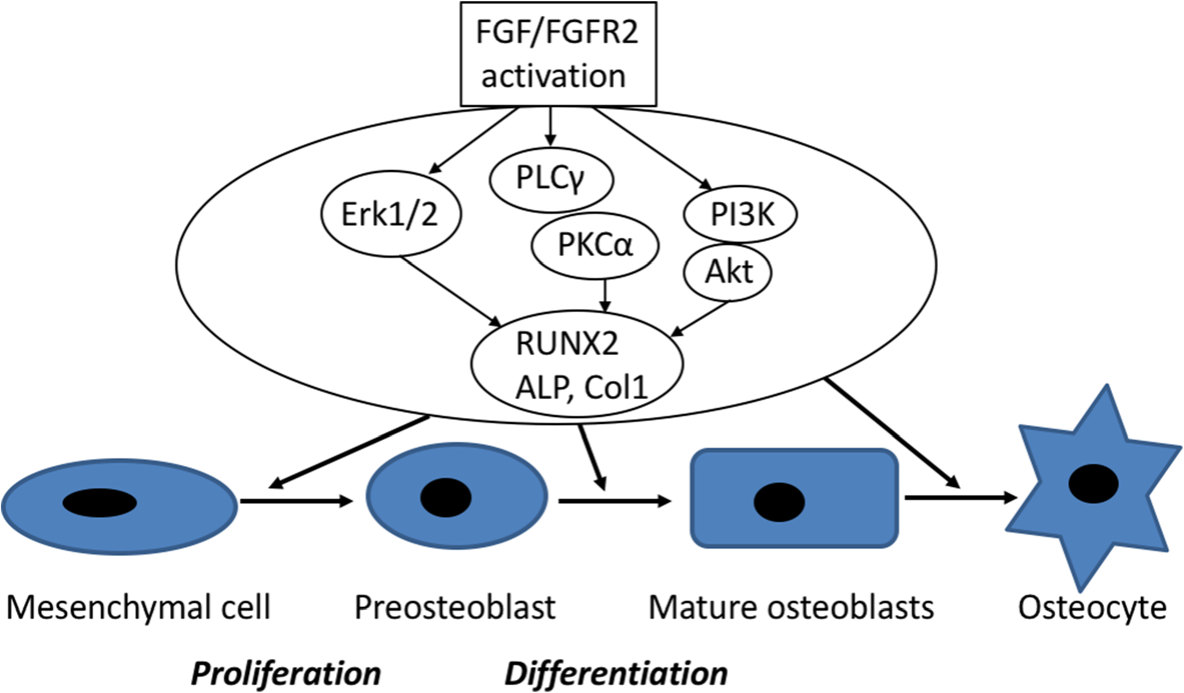
Schematic representation of FGF-FGFR signaling pathways and mechanisms in osteogenesis. The regulation of osteogenesis by FGF and FGFR. FGF/FGFR signaling is an important regulator of osteoblastogenesis, and that control osteoblast replication and differentiation. Activation of FGF and FGFR triggers the activation of ERK1/2 MAPK, PLCγ/PKC, and Akt activity which upregulate osteoblast gene expression and osteogenesis. Abbreviations: FGF, fibroblast growth factor; FGFR, fibroblast growth factor receptors

FGFs are proteins identified from pituitary glands in cows and they are found in most tissues throughout the human body [20, 21]. These GFs have various physiological activities and form a family comprising FGF-1 to FGF-23 [1, 3, 22]. FGF-2, FGF- 9, and FGF-18 were first identified in mesenchymal cells and osteoblasts aggregated in the fetal period during which FGFs play an important role in skeletal development. GFs generally act as systemic or locally circulating molecules of extracellular origin that activate cell surface receptors. The genetic mutations of FGF receptors (FGFRs) lead to various diseases that cause abnormal skeleton formation, such as Pfeiffer, Apert, Crouzon, and Jackson–Weiss syndromes [23]. It must be noted that FGFR3 mutations cause achondroplasia and type II thanatophoric dysplasia, which result in dwarfism secondary to a growth cartilage disorder [20, 21]. This evidence demonstrates that FGF signaling performs an important role in the inhibition of bone and cartilage formation during developmental and growth periods, and its research has drawn much attention within the field of bone metabolism [1, 3, 24]. Furthermore, FGF-2, known as basic FGF, is thought to promote cell proliferation and differentiation through various actions, such as vasoformative processes. FGF-2 contributes to angiogenesis, wound healing, and bone repair. Reportedly, FGF-2 increases the number of osteoblasts and chondroblasts. Furthermore, FGF-2 induces differentiation and proliferation of mesenchymal stem cells, resulting in bone regeneration. The FGFR family has four members, FGFR1 to FGFR4. It has been reported that FGF exerts its action by activating FGFR1–3 and signaling pathways that control cells of osteoblast lineage [1, 3, 22, 24, 25] (Fig. 3).

While FGF-2 exhibits a strong angiogenetic action, it has a short half-life. Tissue regeneration using a GF alone has not been successful because the half-life of the GF is insufficient to sustain biologic activity. Arakawa et al. reported that FGF-2 is susceptible to heat (temperatures over of 37 °C or higher) and proteolytic enzymes such as trypsin. Further, the half-life of FGF in vivo is short, ~ 12 h or less [26]. Therefore, it was essential to develop DDS using appropriate scaffolding that enables the drug to act locally for a defined period of time. Of the various DDS that have been developed, biodegradable gelatin hydrogel incorporating rhFGF-2 has been developed and proceeded successfully in Japan. Thus, FGFs are multifactorial proteins with a wide variety of effects that are expected to be applied clinically for tissue regeneration [1, 3, 22, 24]. In the field of osteoarticular medicine, animal experiments have revealed that the use of gelatin hydrogel increases bone formation and mass in the defective bone area [7–9, 18, 19]. Thus, the gelatin hydrogel serves as scaffolding for cell proliferation, promotes the induction of biological tissue regeneration, and enables continued bioactivity of cell GFs.

### Clinical trial using injectable rhFGF-2 preparation

#### Revascularization treatment for lower limb ischemia and ischemic heart disease

The first clinical study of arterial regenerative medicine using rhFGF-2 for lower limb ischemia (such as Buerger’s disease and arteriosclerosis obliterans) was reported by Marui et al. [10]. Under lumbar anesthesia, gelatin hydrogel granules containing rhFGF-2 were injected into 40 sites at the gastrocnemius muscle of the ischemic limb at a dose of 1 ml per injection (total dose of 40 ml and total rhFGF-2 of 20 μg). The primary efficacy endpoint was the transcutaneous oxygen tension of the affected area, which significantly increased from the pre-administration of the trial drug at 4 and 24 weeks to that after administration, indicating an improvement in the primary efficacy endpoint. Significant improvements were also reported in secondary endpoints, including the 6-min walk distance, cyanosis, clinical symptoms defined by Rutherford’s chronic limb ischemia classification, and symptoms at rest assessed using a pain scale. Although no significant improvement was observed in ischemia-related ulcerations, improvements were reported in three of the four patients with ischemia-related ulcers from baseline to 4- and at 24-week follow-up visits. The drug tested was developed as a means to treat lower limb ischemia that could not be cured with standard treatment methods [27]. For the purpose of that research study, a gelatin hydrogel sheet containing rhFGF-2 was developed for two patients with severe ischemic heart disease requiring coronary artery bypass surgery.

#### High tibial osteotomy

Kawaguchi et al. reported the first prospective multicenter clinical trial of the effect of rhFGF-2 on promoting bone formation [11]. This study included 57 patients (aged 40–74 years) who underwent high tibial osteotomy for knee osteoarthritis and were assigned to either the low- (200 μg), moderate- (400 μg), or high- (800 μg) dosage groups (*n* = 20, 18, 19, respectively). No significant between-group differences were noted for patient characteristics, including sex, age, height, and weight. After osteotomy and fixation, a controlled-release gelatin preparation containing the assigned dose of rhFGF-2 was injected into the osteotomy site, and the wound was closed. At 16 weeks after surgery, bone union was assessed by blinded independent assessors using radiograph; results indicated that rhFGF-2 improved the rate of bone healing in a dose-dependent manner (*P* = 0.035). The time to achieve bone union in 50% of patients in the low-, moderate-, and high-dosage groups was 11.5, 10.1, and 8.1 weeks, respectively; the rate of bone union at 8 and 10 weeks in the high-dosage group was approximately three- and twofold that of the low-dosage group, respectively. Furthermore, rhFGF-2 reduced, in a dose-dependent manner, the time in which patients became pain-free, achieved full weight bearing, and could have the external fixation device removed. Conversely, no significant differences were noted before and after surgery or among groups in serum bone metabolism markers or FGF-2 concentrations. Moreover, during the observation period, anti-FGF-2 and anti-gelatin antibodies could not be detected, and no observed adverse events were found to correlate with dosage. For ethical reasons, the study did not include a carrier-only control group; however, compared with the results of animal experiments, the findings of a clear dose-dependent action indicate that rhFGF-2 has the same bone anabolic action in humans and animals. This study by Kawaguchi et al. [11] was the first study worldwide to clearly demonstrate that the promotion of bone formation by rhFGF-2 can be applied safely and effectively in clinical settings.

#### Tibial fractures

Kawaguchi et al. conducted a randomized, placebo-controlled, double-blind comparative study of rhFGF-2-controlled release gelatin to promote fracture healing in patients using animal models of rhFGF-2-controlled release gelatin [14]. The study included 71 patients aged 20–75 years with Gustilo type I open or closed transverse or short oblique diaphyseal fractures. These patients were treated over a 2-year period at 48 institutions in Japan, did not meet any of the exclusion criteria, and provided consent. The subjects were randomly allocated to one of three groups that received the gelatin hydrogel preparation as follows: placebo (without rhFGF-2), low-dosage (0.8 mg of rhFGF-2), and high-dosage (2.4 mg of rhFGF-2) groups. Immediately after the fixation, the allocated preparation was injected into the fracture site. Bone union was evaluated by standard radiographs every 2 weeks over a 24-week period after administration. These radiographs revealed that the time to bone union was significantly lower in both groups that received rhFGF-2 (*P* = 0.031 and *P* = 0.009 for the low- and high-dosage groups, respectively) than that in the placebo group. Additionally, the number of days to achieve bone union was 28 and 27 days shorter in the low- and high-dosage groups, respectively, than that in the placebo group; no significant difference was noted between the low- and high-dosage groups (*P* = 0.776). Bone union was not achieved after 24 weeks in four patients in the placebo group and in one patient in the low-dosage group, but it was achieved in all the patients in the high-dosage group. There was no significant difference in the incidence of adverse events among the three groups. Therefore, these results demonstrated that the local administration of rhFGF-2 was indeed effective and safe for tibial fractures.

#### Periodontal disease

An exploratory phase II clinical trial [12] and post-treatment survey [13] targeted periodontal disease cases reported by Kitamura et al. in 2008 and 2011, respectively, investigating rhFGF-2 for periodontal disease. The trial was a double-blind, multicenter, collaborative, randomized, placebo-controlled design conducted from 2001 to 2004. The study sample comprised 79 participants, of which 20 received placebo. At the time of undergoing flap surgery with hydroxypropylcellulose (HPC) as the substrate, the participants received either a placebo (0%) or rhFGF-2 at a concentration of either 0.03%, 0.1%, or 0.3%. At 36 weeks, alveolar bone regeneration in the test tooth was evaluated as the primary endpoint. Based on standard radiographic images, the results confirmed that the local administration of an HPC preparation containing 0.3% rhFGF-2 for two- or three-walled periodontal bone defects had a significant effect on periodontal bone regeneration. These findings confirmed that rhFGF has a strong regenerative action in periodontal bones, with no notable safety issues reported.

Long-term progress was subsequently examined using information from medical records based on the details and date of treatment for the test tooth, in which was administered either the assigned concentration of rhFGF-2 or the placebo, and the appearance of symptoms in the test tooth was examined over approximately 8 years from the day of the final clinical trial observation. A survival analysis was performed with events defined as treatment or symptoms that were deemed to result from the exacerbation of periodontitis at the trial drug administration site, and all other events were excluded. Events occurred in 14 patients, and survival analysis revealed that the period until the event onset was significantly longer in the group administered 0.3% rhFGF-2 than in the placebo group that underwent flap surgery alone (generalized Wilcoxon test *P* = 0.0345). Furthermore, no complications regarding the safety of rhFGF-2 administration over the observation period were noted. In November 2016, the Japanese national health insurance price listing was allocated to rhFGF-2, and the drug is presently used widely by dentists.

#### Osteonecrosis of the femoral head

**Fig. 4:**
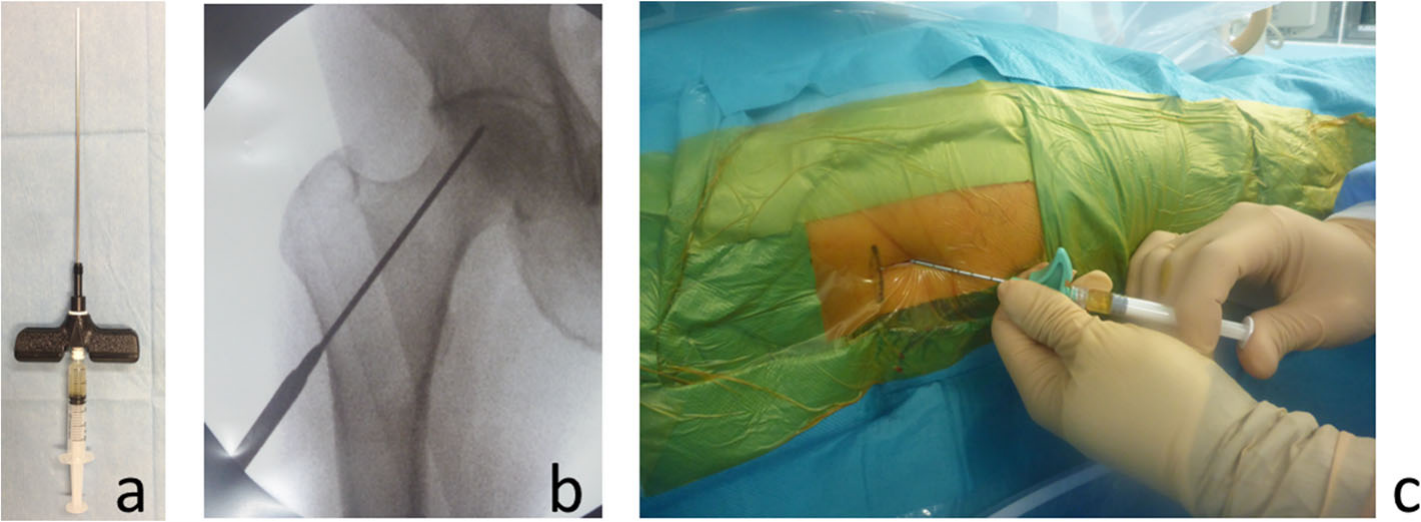
Percutaneous administration of growth factors into the femoral head. Local injection can be percutaneously administered to the target site. This reduces the operating time and damage to surrounding tissue and enables surgery to be minimally invasive. **a** A syringe with long needle available for percutaneous administration. **b** An intraoperative radiographic image reaching to the target site. **c** An intraoperative photograph when pushing the plunger to inject

We reported an exploratory clinical trial of the percutaneous administration of a gelatin hydrogel containing rhFGF-2 for osteonecrosis of the femoral head [17, 28]. The study sample included 10 patients (five male and five female patients with a mean age of 39.8 years) with pre-collapse osteonecrosis (stage ≤ 2) that was induced by steroids in eight and by alcohol in two patients. The primary endpoint was the incidence of adverse events, and the secondary endpoints were the inhibition of femoral head collapse, changes in disease staging, clinical evaluations (visual analog score for pain, Harris hip score, and University of California, Los Angeles activity score), and assessment of bone regeneration in the necrotic area. Surgery was performed under lumbar anesthesia, and a 1-cm incision was made through which a hydrogel containing 800 μg of rhFGF-2 was percutaneously administered (Fig. 4). No problematic adverse events were noted, with the exception of one patient who had a maximum necrotic volume of 70% at the time of surgery and no cases of femoral head collapse or progression of disease staging. The mean operative duration was 18 min, walking was permitted from the day following surgery, and the mean hospital stay was 6 days. All clinical scores were improved 1 year after surgery compared with the preoperative scores. Notably, a subsequent, physician-initiated, multicenter trial including 64 subjects started from January 2016. The 2-year observation period was completed by March 2019, and analyses are underway.

### Bone morphogenic proteins (BMPs)

In 1965, Urist reported BMPs as factors with a capacity for heterotopic bone formation, which are found in the demineralized bone matrix. Since then, BMPs have been better known as bone- and cartilage-inducing factors that promote bone formation in vivo [29]. Data from gene cloning has revealed that BMP exists in nearly 20 isoforms. The TGF-β superfamily is classified into four subgroups comprising BMPs, activin, inhibin, and TGF-β, with various functions, such as development and tissue homeostasis [30]. Among these, BMP-2, BMP-4, BMP -6, and BMP -7 have a strong impact on bone formation. BMPs act on undifferentiated mesenchymal cells and induce runt-related transcription factor 2 (Runx2) expression and osteoblast differentiation. However, in Runx2 knockout cells, evidence that BMPs induce osteoblast differentiation demonstrates the presence of a Runx2 non-dependent pathway. BMP-2 and BMP-7/OP-1 (osteogenic protein) play an important role in the regulation of undifferentiated mesenchymal cells to osteoblasts and also possibly in bone regeneration. Therefore, they have been examined in detail as factors that promote bone formation in various tissues and cells both in vitro and in vivo.

In the field of orthopedic surgery, a kit combining rhBMP-2 and absorbable collagen sponge (bovine type I collagen) is commercially available as a medical device for guiding bone regeneration (InFUSE Bone Graft, Medtronic Sofamor Danek, Inc., Minneapolis, MN, USA). It is primarily used in the treatment of spinal fusion and tibial fractures [31–33] and is used less frequently for osteonecrosis of the femoral head [34–36]. The high-usage rate for spinal fusion increases costs and the device has been associated with adverse events resulting from concentration settings in clinical application and off-label administration [37, 38]. The US Food and Drug Administration (FDA) issued a statement in July 2008 regarding life-threatening complications associated with InFUSE bone grafts. The rhBMP-7/OP-1 (Putty, Stryker, Kalamazoo, MI, USA) has obtained a humanitarian device exemption from the FDA, allowing it to be used for lumbar spine fusion and the treatment of long bone fractures [39, 40].

### Platelet-derived growth factor (PDGF)

PDGFs are produced by osteoblasts and primarily act to promote bone cell proliferation and mesenchymal cell migration simultaneously. Their effect on wound healing is promising, and among the known GFs, the clinical application of PDGFs is extensive and continues to expand. PDGFs exhibit weaker promotion of bone formation than that exhibited by other GFs; however, when combined with IGF-1, it has been found to promote bone formation in the field of dentistry [41]. PDGFs exist as dimeric forms of the polymerized monomers PDGF-A and PDGF-B, with the strongest activity demonstrated by PDGF-BB. PDGFs act on osteoblasts to promote proliferation and collagen production but not osteoblast differentiation [42].

Regranex gel (Ortho-McNeil Pharmaceutical, Raritan, NJ, USA) is the first FDA-approved rhPDGF product (100 μg/g) for the treatment of lower limb diabetic ulcers [43]. It is supplied in multi-use tubes as a non-sterile solution for topical use, containing 15 g of gel. GEM-21S (LYNCH Biologics LLC, Franklin, TN, USA), which is commercially available in the USA, induces periodontal tissue regeneration by combining PDGF-BB and β-tricalcium phosphate (a prosthetic bone material). It thus constitutes an example of the application of cytokines in this field. A randomized controlled trial, which included a 3-year follow-up after surgery, demonstrated that combining 0.3 mg/ml of rhPDGF-BB with a carrier improves the periodontal pocket depth compared to that observed with the carrier alone and achieves clinical attachment levels [44].

### Vascular endothelial growth factor (VEGF)

VEGFs primarily act on endothelial cells as paracrine factors, and their bioactivity promotes angiogenesis, cell division, vascular permeability, and chemotactic activity. Both VEGF and VEGF receptors are expressed in osteoblasts; the addition of VEGF directly promotes osteoblast mineralization, demonstrating that VEGF promotes their differentiation [45]. The production of VEGFs in osteoblasts is enhanced by BMP via the p38 mitogen-activated protein kinase pathway; however, increased VEGF production, induced by BMPs, promotes bone formation by enhancing angiogenesis rather than the mineralization of osteoblasts. VEGFs are clinically applied to improve angiogenesis and tissue ischemia in diseases affecting the lower limbs [46].

### Insulin-like growth factor (IGF)

IGF-1 was initially identified as an insulin-like growth factor; however, it has recently been found to exert various effects on cell protection and proliferation. IGF increases the signaling needed for cell survival, reduces unnecessary active oxygen, and inhibits apoptosis [47]. Furthermore, it increases cellular energy metabolism, promotes cell growth and dopamine neurotransmission in a functional manner, and consequently contributes to the regeneration of nerve cells [48]. IGFs include IGF-1 and –H, and although IGF-II is predominantly expressed in the fetal period, IGF-1 appears to have a greater role in growth and development after birth. IGF-1 is primarily produced by the liver in a growth hormone-dependent manner. However, in bone tissues, IGF-1 is produced by osteoblasts and acts as a local GF through autocrine/paracrine activity and accumulates abundantly in the bone matrix. In the field of otorhinolaryngology, gelatin hydrogel containing IGF-1 is clinically applied for the treatment of sudden deafness [15, 16].

## Discussion

In the 1970s, developments in recombination DNA technology enabled the refinement of proteins with various physiological activities, such as interferon and granulocyte colony-stimulating factor, which have been used as pharmaceuticals in clinical practice. By contrast, cell GFs are proteins that act in small concentrations, are classified as locally acting cytokines that cannot pass through the lipid bilayer of cell membranes, and instead act by binding to receptor proteins that pass through cell membranes [1, 3–6].

There are several possible reasons for the development of regenerative medicine using GFs. First, regenerative medicines, such as gene and cell therapy, have been attempted; however, while many studies have reportedly suggested that they are useful procedures, several associated problems, including the short- and long-term safety of genetic materials such as viruses and plasmids, exist. Second, while cell transplantation using autologous cells has excellent safety, a highly invasive harvesting process is required to ensure a sufficient number of cells. Additionally, no consensus has been reached regarding the isolated cell type and number of transplanted cells. Therefore, recombinant technology has also been developed, and the application of GFs in regenerative medicine has advanced. Furthermore, the advent of carriers that are capable of controlled release has promoted basic research [1–3]. If target proteins could be developed to act locally and effectively for a defined period, this could aid in avoiding not only time-related and ethical restrictions but also economical barriers, such as those associated with cell culture and gene transfer, enabling the implementation of simplified regenerative medicines. The ideal DDS would characteristically involve an adjustable period for hydrogel biodegradation of several days to months during which proteins would be locally released in a predominantly constant, controlled manner, exhibiting continuous physiological activity. Of the various carriers studied, natural polymers including collagen, gelatin, fibrinogen, and hyaluronic acid have gained wide attention as scaffold-based DDS. The main reason for this is that Se carriers are often soluble in water and are relatively harmless to the bioactivity of GFs. Table 1 of the present review shows that in past trials of gelatin hydrogels, there were no problematic adverse events, which allowed incorporating GFs as effective regenerative medicine approaches. Although all topically applied products have the risk of being irritating or causing allergic contact dermatitis, the past clinical trials using gelatin hydrogels set the exclusion criteria of hypersensitivity to gelatin for reducing the adverse event. Gelatin is used in several food products and as regulating material that is clinically applied; it turns into non-toxic amino acids after its breakdown in the body.

Among the many identified GFs, research and development of rhFGF-2 in Japan have progressed considerably mainly as a result of carriers, such as hydrogels, which allow a controlled release of the GF [7–9, 18, 19]. Accordingly, rhFGF-2 has already been developed into products for bedsores (Fiblast Spray, Kaken Pharmaceuticals Co., Ltd. Tokyo, Japan) [49, 50]. Based on the research and development of the rhFGF-2 product, several in vitro studies were underway to determine the mechanisms underlying the promotion of bone formation by FGF-2 observed in vivo from the perspective of osteoblastic cell proliferation, differentiation, and matrix synthesis [20, 21]. Kawaguchi et al. demonstrated that FGF-2 has a strong promoting action on cell proliferation, especially on undifferentiated osteoblast precursors and bone marrow stromal cells [51]. By contrast, the effect of FGF-2 on osteoblastic cell differentiation and matrix synthesis, particularly on collagen synthesis, appears consistent in terms of inhibition. Therefore, the role of FGFs in fracture healing primarily involves the promotion of undifferentiated mesenchymal cell proliferation, indicating that subsequent cell differentiation and matrix synthesis are promoted by other GFs such as cytokine cascades, including TGF-β and BMPs. Indeed, FGF-2 promotes the synthesis of TGF-β, IGF, and their binding proteins. In the first human clinical trial using the injectable rhFGF-2, Kawaguchi et al. reported single injections of rhFGF-2-impregnated hydrogel at doses of 200, 400, and 800 μg in cut surfaces of the tibia, which resulted in a rapid and dose-dependent synostosis [11]. Furthermore, Kawaguchi et al. reported the safety and efficacy of the clinical use of gelatin hydrogel containing high-dose rhFGF-2 (2.4 mg) [14]. Based on these reports and the positive results of clinical trials for osteonecrosis and periodontitis [12, 13, 17], the local injection of a gelatin hydrogel impregnated with rhFGF-2 is considered safe and feasible in the field of orthopedics and dentistry. Nevertheless, the clinical application of FGF is not limited to the field of osteoarticular medicine. In fact, FGF gelatin hydrogel has also been applied in vascular surgery [10, 27]. Nakagawa et al. reported that the local application of a gelatin hydrogel containing IGF-1 was effective for the treatment of sudden deafness [15, 16].

In the field of bone regeneration, GFs, such as BMP-2, OP-1/BMP-7, IGF, VEGF, PDGF, and FGF-2, have synergistic effects and the consecutive signaling improved bone healing within animal models, and thus, have been widely utilized. However, due to the important safety concerns, commercially available GF-containing products remain limited. In Europe and the USA, enhanced bone repair has also been demonstrated in the clinic following European Medicines Agency and FDA approval of rhBMP-2 [31–38], rhBMP-7/OP-1 [39, 40], and rhPDGF [43, 44]. In Japan, the clinical use of rhFGF-2 for skin ulcers [49, 50] and periodontitis [12, 13] was approved by Pharmaceuticals and Medical Devices Agency. In the present study, Table 2 demonstrated that the combination of GF and scaffolds differed. This point might be closely related to the background of development from basic experiments and authorization systems of drugs or medical devices. However, while BMP products have been widely used in Europe and the USA, they are not approved in Japan. The same explanation is fitting for FGF-2, PDGF, and VEGF. There are concerns that off-label use of rhBMP-2 or rhBMP-7 may have irreversible complications including excessive bone formation, paralysis (spinal cord or nerve compression), severe pain, and even death [37, 38]. The US FDA issued a statement regarding life-threatening complications associated with the off-label use of both rhBMP products. As a result, the latter manufacture making rhBMP-7 was forced to pay for its illegal promotion of the off-label use with the tricalcium phosphate scaffold. Therefore, rhBMP products have not yet become standard-of-care therapies in regenerative medicine. We think that these problematic complications might not occur in the clinical applications using the bioabsorbable natural agents, such as the gelatin hydrogel.

**Table 2:** Commercially available growth factor-containing products for local application

Product name, company, country, year of approval	Target	Growth factor	Carrier	Appearance	Injection use
Regranex®, Ortho-McNeil Pharmaceutical, USA, 1997	Skin ulcer	PDGF-BB	Hydrogel	Gel	Possible, but its use for coating a skin
InFUSE®, Medtronic, USA, 2002	Spinal fusion, fracture, bone graft	BMP-2	Bovine bone collagen	Sheet	No, use in open surgery
Putty®, Stryker, USA, 2004	Spinal fusion, fracture	BMP-7/OP-1	Bovine bone collagen	Gel	Yes
GEM-21S®, LYNCH Biologics LLC, USA, 2005	Periodontitis	PDGF-BB	β-TCP	Hard granules	No
Regroth®, Kaken pharmaceuticals, Japan, 2016	Periodontitis	FGF-2	Hydroxypropylcellulose	Gel	Yes, injectable kit for local administration

*PDGF* platelet-derived growth factor, *BMP* bone morphogenetic proteins, *OP* osteogenic protein, *FGF* fibroblast growth factor, *TCP* tricalcium phosphate

By comparison with cell transplantation and gene therapy, patient administration of gelatin hydrogel is extremely simple and cost effective, with excellent feasibility. The greatest advantage of injectable GF is the bioabsorbable properties of the gelatin hydrogel, its minimal invasiveness, and high safety. The controlled release of GFs using an injectable gelatin hydrogel presents new possibilities that compensate for shortcomings in conventional regenerative therapy. In the future, long-term therapeutic effects, appropriate treatment duration, and selection of target conditions should be examined in further large-scale clinical trials. However, through ongoing collaborative clinical and basic research, we hope to elucidate the role of treatment using injectable GFs in relation to conventional treatment methods and to develop the approaches further as emerging therapies.

## Conclusions

The clinical application of injectable GFs using natural polymers, such as the gelatin hydrogel, is considered safe and feasible for tissue regeneration and will probably be developed further and gain even greater popularity as a novel medical approach applicable to various fields. Injectable GF treatment can reduce the operating time and damage to surrounding tissue and enables surgery to be minimally invasive.

## Data Availability

Not applicable.

## Abbreviations

BMP: Bone morphogenetic proteins
DDS: Drug delivery system
FDA: Food and Drug Administration
FGF: Fibroblast growth factor
FGFR: Fibroblast growth factor receptor
GF: Growth factor
IGF: Insulin-like growth factor
NGF: Nerve growth factor
PDGF: Platelet-derived growth factor
rh: Recombinant human
VEGF: Vascular endothelial growth factor

## References

[1] Mitchell AC, Briquez PS, Hubbell JA, Cochran JR (2016). Engineering growth factors for regenerative medicine applications. Acta Biomater.

[2] Lorentz KM, Yang L, Frey P, Hubbell JA (2012). Engineered insulin-like growth factor-1 for improved smooth muscle regeneration. Biomaterials.

[3] Lee K, Silva EA, Mooney DJ (2011). Growth factor delivery-based tissue engineering: general approaches and a review of recent developments. J R Soc Interface.

[4] Hamburger V (1993). The history of the discovery of the nerve growth factor. J Neurobiol.

[5] Heldin CH, Westermark B (1984). Growth factors: mechanism of action and relation to oncogenes. Cell.

[6] Folkman J, Klagsbrun M (1987). Angiogenic factors. Science.

[7] Tabata Y, Yamada K, Miyamoto S, Nagata I, Kikuchi H, Aoyama I (1998). Bone regeneration by basic fibroblast growth factor complexed with biodegradable hydrogel. Biomaterials.

[8] Tabata Y, Nagano A, Ikada Y (1999). Biodegradation of hydrogel carrier incorporating fibroblast growth factor. Tissue Eng.

[9] Tabata Y, Hijikata S, Ikada Y (1994). Enhanced vascularization and tissue granulation by basic fibroblast growth factor impregnated in gelatin hydrogels. J Control Release.

[10] Marui A, Tabata Y, Kojima S, Yamamoto M, Tambara K, Nishina T (2007). A novel approach to therapeutic angiogenesis for patients with critical limb ischemia by sustained release of basic fibroblast growth factor using biodegradable gelatin hydrogel: an initial report of the phase I-IIa study. Circ J.

[11] Kawaguchi H, Jingushi S, Izumi T, Fukunaga M, Matsushita T, Nakamura T (2007). Local application of recombinant human fibroblast growth factor-2 on bone repair: a dose-escalation prospective trial on patients with osteotomy. J Orthop Res.

[12] Kitamura M, Nakashima K, Kowashi Y, Fujii T, Shimauchi H, Sasano T (2008). Periodontal tissue regeneration using fibroblast growth factor-2: randomized controlled phase II clinical trial. PLoS One.

[13] Kitamura M, Akamatsu M, Machigashira M, Hara Y, Sakagami R, Hirofuji T (2011). FGF-2 stimulates periodontal regeneration: results of a multi-center randomized clinical trial. J Dent Res.

[14] Kawaguchi H, Oka H, Jingushi S, Izumi T, Fukunaga M, Sato K (2010). A local application of recombinant human fibroblast growth factor 2 for tibial shaft fractures: a randomized, placebo-controlled trial. J Bone Miner Res.

[15] Nakagawa T, Sakamoto T, Hiraumi H, Kikkawa YS, Yamamoto N, Hamaguchi K (2010). Topical insulin-like growth factor-1 treatment using gelatin hydrogels for glucocorticoid-resistant sudden sensorineural hearing loss: a prospective clinical trial. BMC Med.

[16] Nakagawa T, Kumakawa K, Usami SI, Hato N, Tabuchi K, Takahashi M (2014). A randomized controlled clinical trial of topical insulin-like growth factor-1 therapy for sudden deafness refractory to systemic corticosteroid treatment. BMC Med.

[17] Kuroda Y, Asada R, So K, Yonezawa A, Nankaku M, Mukai K (2016). A pilot study of regenerative therapy using controlled release of rhFGF-2 for patients with precollapse osteonecrosis of the femoral head. Int Orthop.

[18] Yamamoto M, Takahashi Y, Tabata Y (2006). Enhanced bone regeneration at a segmental bone defect by controlled release of bone morphogenetic protein-2 from a biodegradable hydrogel. Tissue Eng.

[19] Igai H, Chang SS, Gotoh M, Yamamoto Y, Misaki N, Okamoto T (2006). Regeneration of canine tracheal cartilage by slow release of basic fibroblast growth factor from gelatin sponge. ASAIO J.

[20] Ornitz DM, Marie PJ (2015). Fibroblast growth factor signaling in skeletal development and disease. Genes Dev.

[21] Teven CM, Farina EM, Rivas J, Reid RR (2014). Fibroblast growth factor (FGF) signaling in development and skeletal diseases. Genes Dis.

[22] Marie PJ, Miraoui H, Sévère N (2012). FGF/FGFR signaling in bone formation: progress and perspectives. Growth Factors.

[23] Muenke M, Schell U, Hehr A, Robin NH, Losken HW, Schinzel A (1994). A common mutation in the fibroblast growth factor l gene in Pfeiffer syndrome. Nat Genet.

[24] Marie PJ (2012). Fibroblast growth factor signaling controlling bone formation: an update. Gene.

[25] Su N, Jin M, Chen L (2014). Role of FGF/FGFR signaling in skeletal development and homeostasis: learning from mouse models. Bone Res.

[26] Arakawa T, Prestrelski SJ, Kenney WC, Carpenter JF (1993). Factors affecting short-term and long-term stabilities of proteins. Adv Drug Deliv Rev.

[27] Marui A, Doi K, Tambara K, Sakakibara Y, Ueyama K, Iwakura A, Mori H, Matsuda H (2005). Basic fibroblast growth factor and angiogenesis. Cardivascular regeneration therapies using tissue engineering approaches.

[28] Kuroda Y, Matsuda S, Akiyama H (2016). Joint-preserving regenerative therapy for patients with early-stage osteonecrosis of the femoral head. Inflamm Regen.

[29] Urist MR (1965). Bone: formation by autoinduction. Science.

[30] Sanchez-Duffhues G, Hiepen C, Knaus P, Ten Dijke P (2015). Bone morphogenetic protein signaling in bone homeostasis. Bone.

[31] Carreira AC, Lojudice FH, Halcsik E, Navarro RD, Sogayar MC, Granjeiro JM (2014). Bone morphogenetic proteins: facts, challenges, and future perspectives. J Dent Res.

[32] Riedel GE, Valentin-Opran A (1999). Clinical evaluation of rhBMP-2/ACS in orthopedic trauma: a progress report. Orthopedics.

[33] Govender S, Csimma C, Genant HK, Valentin-Opran A, BESTT study group (2002). Recombinant human bone morphogenetic protein-2 for treatment of open tibial fractures: a prospective, controlled, randomized study of four hundred and fifty patients. J Bone Joint Surg.

[34] Lieberman JR, Conduah A, Urist MR (2004). Treatment of osteonecrosis of the femoral head with core decompression and human bone morphogenetic protein. Clin Orthop Relat Res.

[35] Mont MA, Jones LC, Einhorn TA, Hungerford DS, Reddi AH (1998). Osteonecrosis of the femoral head—potential treatment with growth and differentiation factors. Clin Orthop Relat Res.

[36] Papanagiotou M, Malizos KN, Vlychou M, Dailiana ZH (2014). Autologous (non-vascularised) fibular grafting with recombinant bone morphogenetic protein-7 for the treatment of femoral head osteonecrosis: preliminary report. Bone Joint J.

[37] Woo EJ (2012). Adverse events reported after the use of recombinant human bone morphogenetic protein 2. J Oral Maxillofac Surg.

[38] Woo EJ (2013). Adverse events after recombinant human BMP2 in nonspinal orthopaedic procedures. Clin Orthop Relat Res.

[39] Vaccaro AR, Lawrence JP, Patel T, Katz LD, Anderson DG (2008). The safety and efficacy of OP-1 (rhBMP-7) as a replacement for iliac crest auto- graft in posterolateral lumbar arthrodesis: a long-term (>4 years) pivotal study. Spine.

[40] Vaccaro AR, Whang PG, Patel T, Phillips FM, Anderson DG (2008). The safety and efficacy of OP-1 (rhBMP-7) as a replacement for iliac crest autograft for posterolateral lumbar arthrodesis: minimum 4-year follow-up of a pilot study. Spine J.

[41] Lynch SE, Williams RC, Poison AM, Howell TH, Reddy MS, Zappa UE (1989). A combination of platelet-derived and insulin-like growth factors enhances periodontal regeneration. J Clin Periodontol.

[42] Canalis E (2003). Primer on the metabolic bone disease and disorders of mineral metabolism.

[43] U.S. Food and Drug Administration (2008). Safety warning on becaplermin in Regranex®.

[44] Ridgway HK, Mellonig JT, Cochran DL (2008). Human histologic and clinical evaluation of recombinant human platelet-derived growth factor and betatricalcium phosphate for the treatment of periodontal intraosseous defects. Int J Periodontics Restorative Dent.

[45] Horiuchi N (2004). Vascular endothelial cells and osteoblasts. Clin Calcium.

[46] Isner JM, Pieczek A, Schainfeld R, Blair R, Haley L, Asahara T (1996). Clinical evidence of angiogenesis after arterial gene transfer of phVEGF165 in patients with ischemic limb. Lancet.

[47] Emtage P, Vatta P, Arterburn M, Muller MW, Park E, Boyle B, Hazell S, Polizotto R, Funk WD, Tang YT (2006). IGFL: a secreted family with conserved cysteine residues and similarities to the IGF superfamily. Genomics.

[48] Niu T, Rosen CJ (2005). The insulin-like growth factor-I gene and osteoporosis: a critical appraisal. Gene.

[49] Okumura M, Okuda T, Nakamura T, Yajima M (1996). Acceleration of wound healing in diabetic mice by basic fibroblast growth factor. Biol Pharm Bull.

[50] Tanaka E, Ase K, Okuda T, Okumura M, Nogimori KB (1996). Mechanism of acceleration of wound healing by basic fibroblast growth factor in genetically diabetic mice. Biol Pharm Bull.

[51] Kawaguchi H, Pilbeam CC, Gronowicz G, Abreu C, Fletcher BS, Herschman HR (1995). Transcriptional induction of prostaglandin G/H synthase-2 by basic fibroblast growth factor. J CIin Invest.

